# Supplementary material for: The causal relationship between the human gut microbiota and pyogenic arthritis: a Mendelian randomization study
Source: Front Cell Infect Microbiol. 2024 Nov 26;14:1452480. doi: 10.3389/fcimb.2024.1452480 (PMC11629706; doi:10.3389/fcimb.2024.1452480)
Supplement: Supplementary file 1 [file DataSheet1.zip › mendelian test/ebi-a-GCST90027563.csv_leave_one_out.pdf]

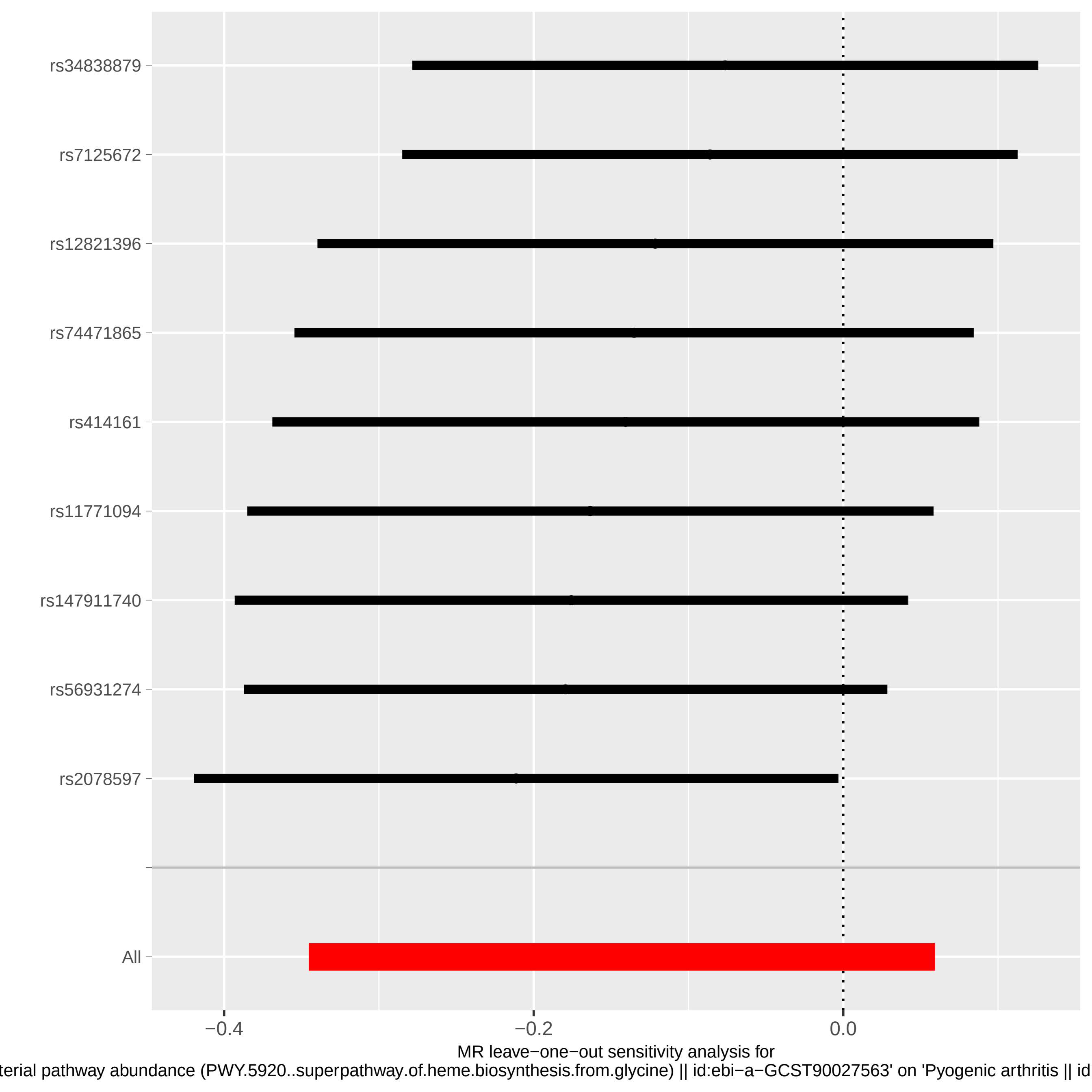

rs34838879
rs7125672
rs12821396
rs74471865
rs414161
rs11771094
rs147911740
rs56931274
rs2078597
All
−0.4
−0.2
0.0
MR leave−one−out sensitivity analysis for
terial pathway abundance (PWY.5920..superpathway.of.heme.biosynthesis.from.glycine) || id:ebi−a−GCST90027563' on 'Pyogenic arthritis || id
